# Immunometabolism in the pathogenesis of vitiligo

**DOI:** 10.3389/fimmu.2022.1055958

**Published:** 2022-11-10

**Authors:** Chen Lyu, Yonghu Sun

**Affiliations:** Shandong Provincial Hospital for Skin Diseases & Shandong Provincial Institute of Dermatology and Veneorology, Shandong First Medical University & Shandong Academy of Medical Sciences, Jinan, China

**Keywords:** vitiligo, immunometabolism, oxidative stress, glucose metabolism, lipid metabolism, immunotherapy

## Abstract

Vitiligo is a common depigmenting skin disorder characterized by the selective loss of melanocytes. Autoimmunity, genetic, environmental, and biochemical etiology have been proposed in vitiligo pathogenesis. However, the exact molecular mechanisms of vitiligo development and progression are unclear, particularly for immunometabolism. Sporadic studies have suggested mitochondrial dysfunction, enhanced oxidative stress, and specific defects in other metabolic pathways can promote dysregulation of innate and adaptive immune responses in vitiligo. These abnormalities appear to be driven by genetic and epigenetic factors modulated by stochastic events. In addition, glucose and lipid abnormalities in metabolism have been associated with vitiligo. Specific skin cell populations are also involved in the critical role of dysregulation of metabolic pathways, including melanocytes, keratinocytes, and tissue-resident memory T cells in vitiligo pathogenesis. Novel therapeutic treatments are also raised based on the abnormalities of immunometabolism. This review summarizes the current knowledge on immunometabolism reprogramming in the pathogenesis of vitiligo and novel treatment options.

## Introduction

*Vitiligo* is an autoimmune skin disease characterized by skin pigmentation loss due to functional melanocyte dysregulation. Many studies suggest that vitiligo is associated with insulin resistance, lipid abnormalities, and other metabolic disorders (1–3). Vitiligo affects approximately 0.5% to 2% of the world’s population (4). Various mechanisms have been proposed to explain the destruction of melanocytes in vitiligo, including genetics, autoimmune response, oxidative stress, and the production of inflammatory mediators (5). However, the exact pathogenesis of immunometabolism abnormalities in vitiligo is not fully understood. In 2016, vitiligo was further classified into three categories: nonsegmental vitiligo (NSV), segmental vitiligo (SV), and undetermined/unclassified (6). NSV is the most common type and has been reclassified as a systemic rather than a depigmented skin disease (7). Regulation of the innate immune response and B-cell differentiation and their activation was demonstrated in NSV. Especially, NSV was reported to be more pronounced than SV (8, 9). Several systemic metabolic disorders suggest a close association with NSV, such as type I diabetes, atherosclerotic cardiovascular disease, and metabolic syndrome (10–12).

Metabolic reprogramming is the modification of different metabolic processes to regulate the function and phenotype of immune cells to meet the regulation and execution of immune functions (13). The focus is on the effects of different metabolic pathways on immune cell activation, differentiation, and function, including glucose metabolism and lipid metabolism (14). Metabolic reprogramming of innate and adaptive immune elements might contribute to the disturbed immune homeostasis in vitiligo. The translational implications of these findings are enormous and may lead to novel therapeutic targets.

## Oxidative stress

### The reciprocal action of reactive oxygen species and the immune system in vitiligo

**Figure 1:**
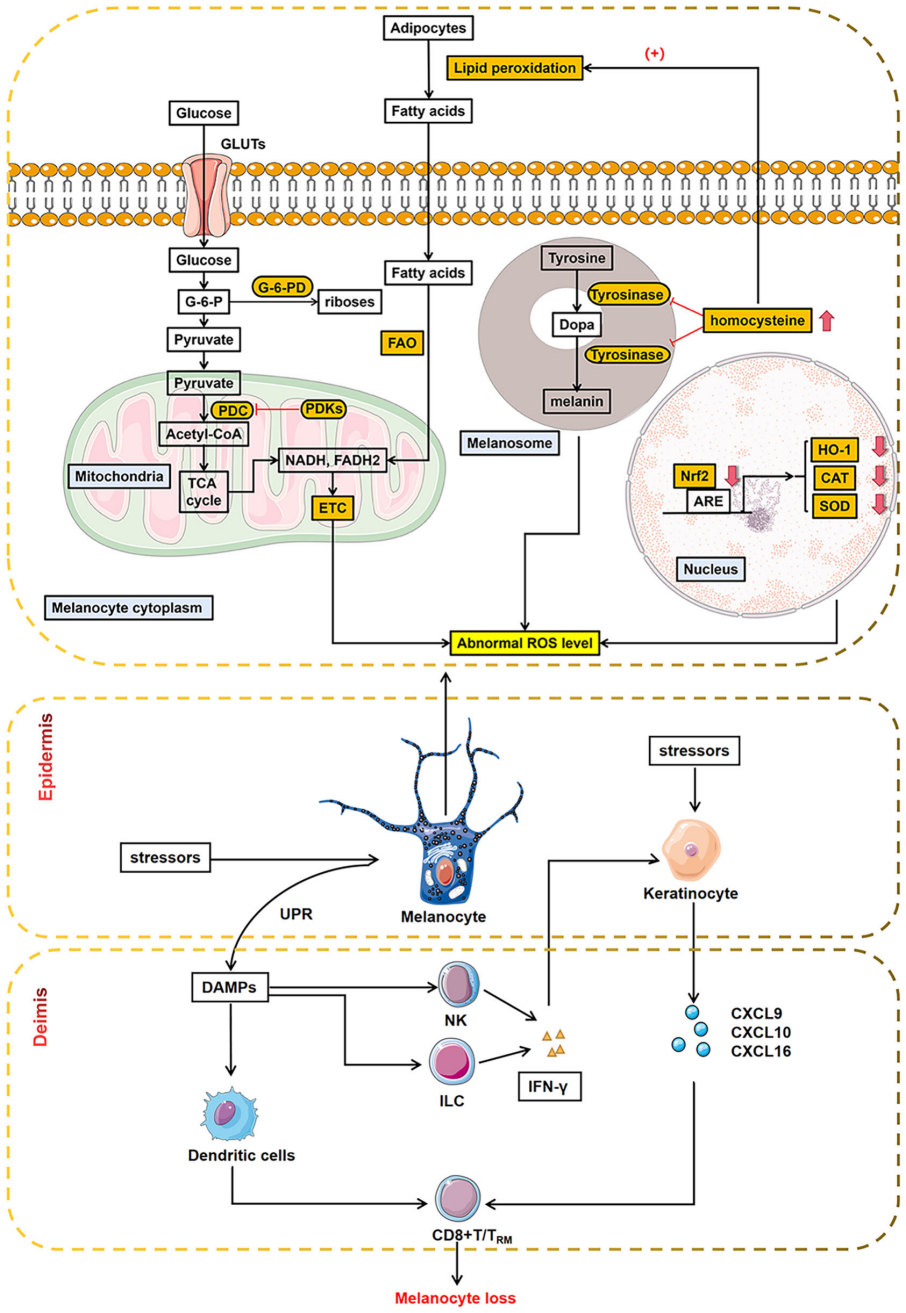
Abnormal metabolic processes in melanocytes during oxidative stress. Oxidative stress triggered abnormal glycolipid metabolism within melanocytes, disrupting metabolic pathways. Melanocytes with mitochondrial disorders, abnormal melanin biosynthesis, and dysregulated metabolic pathway produce abnormal ROS, exacerbating oxidative stress. The orange-boxed portion of the figure shows outlier segments, as reported in recent literature. In addition, melanocytes interact with different immune cells in innate and adaptive immunity, leading to melanocyte loss ultimately. ARE, antioxidant response element; CAT, catalase; HO-1, heme oxygenase-1; Nrf2, nuclear factor E2-related factor 2; ROS, reactive oxygen species; SOD, superoxide dismutase; GLUTs, glucose transporters; PDC, pyruvate dehydrogenase complex; PDKs, pyruvate dehydrogenase kinase; ETC, electron transport chain; FAO, fatty acid oxidation; G-6-PD, glucose-6-phosphate dehydrogenase; UPR, unfolded protein response; DAMPs, damage-associated molecular patterns; NK, natural killer cells; ILC, innate lymphoid cells; IFN-γ, inteferon-γ; CXCL9, C-X-C motif chemokine ligand 9; CXCL10, C-X-C motif chemokine ligand 10; CXCL16, C-X-C motif chemokine ligand 16.

Reactive oxygen species (ROS) induced oxidative stress triggers a wide range of abnormal organelle functions, disrupts metabolic pathways, and impairs defense mechanisms against oxidant shocks. It plays a crucial role in cellular events such as inflammatory responses, altered cell growth, and apoptosis (**Figure 1**) (15–20).

The role of alterations in the antioxidant system in the pathogenesis of vitiligo has been extensively studied (21). Low levels of catalase (CAT), glutathione peroxidase (GPx), glucose-6-phosphate dehydrogenase (G6PD), and superoxide dismutase (SOD) have been demonstrated in the epidermis and blood of vitiligo patients (22–29). In addition, polymorphisms in the *CAT* gene and reduced catalase activity have also been associated with several metabolic diseases, such as diabetes, hypertension, and peroxisomal/peroxisomal diseases (30–39). All of the above etiologies can lead to chronic oxidative stress (OS) state that causes melanocytes to be programmed for cellular senescence. Senescent melanocytes exhibit a specific “senescence-associated secretory phenotype (SASP),” which recruits immune cells to remove “senescent melanocytes” by expressing various cytokines (40).

On the other hand, the chronic OS will produce an excessive accumulation of ROS, which in turn induces the production of damage-associated molecular patterns (DAMPs) and the release of melanosomal antigens that activate innate immunity (41). Natural killer (NK) cells and innate lymphoid cells (ILC) secret interferon-γ (IFN-γ) to induce expression of C-X-C motif chemokine receptor (CXCR)3B on melanocyte surface and release of C-X-C motif chemokine ligand 9 (CXCL9), C-X-C motif chemokine ligand 10 (CXCL10), and C-X-C motif chemokine ligand 11 (CXCL11) from keratinocyte and melanocyte. CXCL10 then activates CXCR3B and triggers the apoptosis of melanocytes (42). What’s more, melanocyte specific CD8^+^ T cells are activated through HSP70i activation and antigen presentation by mature dendritic cells (43, 44). In response to cytokine and chemokine stimuli, CD8^+^ T cells are recruited to induce melanocyte apoptosis (45, 46). Furthermore, dysfunctional regulatory T cells (Tregs) in patients with active vitiligo contribute to pathogenesis by impairing the suppressive activity of CD8^+^ T cells (47, 48).

These findings suggest that oxidative stress plays a vital role in the pathogenesis of vitiligo. Abnormalities in ROS serve as a bridge between oxidative stress and immune response.

### Interaction between mitochondrial dysfunction and oxidative stress in vitiligo

Mitochondrial dysfunction is considered essential in losing redox homeostasis (high spontaneous ROS production and lack of antioxidant network) in vitiligo (49–51). A study by Bhattiet et al. mentioned that oxidative stress leads to ROS deposition in cells and tissues by interacting with mitochondrial and cellular components such as DNA, proteins, and lipids leading to mitochondrial dysfunction (52). Dysfunctional mitochondria in melanocytes and CD8^+^ T cells are significant for the induction of melanocyte apoptosis.

Studies of melanocytes and peripheral blood mononuclear cells (PBMCs) from vitiligo patients have shown a defect in complex I formation (51, 53). Due to low concentrations and abnormal distribution of cardiolipin of the mitochondrial electron transport chain (mETC), the stability of the mitochondrial supercomplex was impaired, and the level of glycolytic phosphorylation was decreased (54). Consequently, the production of ATP is reduced, and mitochondrial ROS are highly emitted. Interestingly, cardiolipin replacement rescues normal mitochondrial function in vitiligo patients (54). However, more studies are needed to determine the reason for the low concentration of mitochondrial cardiolipin in melanocytes and CD8^+^ T cells and how to restore average concentrations of this lipid *in vivo*.

In vitiligo patients, NADPH oxidase isoform 4 (NOX4) in the mitochondria produces a large amount of H_2_O_2_ (55). However, higher NOX4 activity depletes NADPH molecules used as cofactors for CAT, GPX, and thioredxin reductase (TrxR) in H_2_O_2_ buffer activity (56). In addition, excess H_2_O_2_ impairs CAT, GPX, and TrxR buffering action. Meanwhile, it causes mitochondrial swelling and changes in mitochondrial morphology upon loss of calcium metabolism. Besides inhibiting the mitochondrial autophagy process, morphological altered mitochondrial hyperactivated P53 and enhanced SOD enzyme activity (28, 57). Thus, the highly damaged mitochondria continue to produce large amounts of ROS, which induce melanocyte apoptosis *via* mitochondrial cytochrome c release or massive recruitment of CD8^+^ T cells.

### Abnormalities in endoplasmic reticulum stress

In addition to serving as essential sites for the synthesis, folding, modification, and trafficking of proteins, the endoplasmic reticulum (ER) senses cellular stress and regulates cell survival or death (58). Under unstressed conditions, three ER stress sensors (inositol-requiring enzyme 1α (IRE1α), PKR like endoplasmic reticulum kinase (PERK), and activating transcription factor 6 (ATF6) (59–62)) mainly bind to GRP78(78 kDa glucose-regulated protein), helping to keep it inactive. In the presence of differentiation stimuli, abnormal protein folding in the ER activates the unfolded protein response (UPR) signaling, which can be alleviated by global translational attenuation, induction of molecular chaperones, misfolded protein degradation by endoplasmic reticulum-associated degradation (ERAD), and apoptosis (63). Accumulation of misfolded proteins increases the production of BiP/GRP78, which initiates *XBP1* splicing and induces ATF6 signaling (64, 65).

As the primary immune cells in autoimmunity in vitiligo, the differentiation, proliferation, and homeostasis of CD8^+^ T cells are also influenced by UPR signaling. CD4^−^/CD8 ^−^ progenitor T cells do not display the UPR but greatly increase it during maturation as CD4^+^/CD8^+^ T cells. After differentiation into CD4^+^ T cells, the UPR is suppressed again (64). Infection of mice with lymphocytic choriomeningitis virus (LCMV) leads to upregulation of spliced and unspliced XBP1, further enhancing CD8^+^ T cell differentiation (65). In addition, the UPR of ER stress signaling was activated during epidermal keratinocyte differentiation (66–68). C-X-C motif chemokine ligand 16 (CXCL16) levels are enhanced by IRE1α/XBP1s signaling in stressed keratinocytes, which are involved in recruiting CD8^+^ T cells to vitiligo lesions (69).

ER stress has also been implicated in the regulation of Tregs. Human Tregs clones produced increased interleukin-10 (IL-10) when treated with thapsigargin, an activator of ER stress and UPR, in an eIF2α phosphorylation-dependent manner (70). Loss of ATF4 leads to an increase in Forkhead box P3 (Foxp3) mRNA expression in mouse CD4^+^ T cells differentiated under regulatory conditions in a high oxidative environment (71). Recently, nuclear factor of activated T cells (NFAT) and Foxp3 levels have been reported to be reduced in Tregs from patients with generalized vitiligo, which may impair Tregs function and decrease IL-10 and cytotoxic T-lymphocyte-associated antigen-4 (CTLA4) levels (72–74). However, the exact mechanism between Tregs and ER stress in the pathogenesis of vitiligo remains to be investigated.

What’s more, prolonged ER stress and a defective UPR may lead to the activation of inflammatory transcriptional programs and the release of proinflammatory cytokines, generating further ER stress and oxidative stress. Notably, UPR-induced autophagy is speculated to stimulate the production of exosomes and soluble inflammatory signals that are proinflammatory and promote autoimmunity (46). However, autophagy is essentially a protective response in the face of cellular stress, and defective autophagy increases melanocyte sensitivity to oxidative stress (75). The effects of ROS also extend to other macromolecules in the ER, such as calreticulin (CRT). Calreticulin (CRT), a ubiquitous ER protein regulating intracellular Ca^2+^ homeostasis, was positively correlated with the lesion size and duration of vitiligo in patients (44). Zhang et al. reported that oxidative stress drove the redistribution of CRT from the ER lumen to the cell membrane, thereby improving the immunogenicity of stressed melanocytes and inducing apoptosis (76). In addition to the release of proinflammatory cytokines, melanocyte apoptosis may lead to the release of misfolded/unfolded proteins that have the potential to act as autoantigens and may be recognized by immune cells as DAMPs. Antigen-presenting cells (APCs) may process and present altered proteins/peptides, generating novel epitopes and activating target B and T cells, leading to an autoimmune response against melanocytes (44, 76).

The UPR activated by ER stress plays a crucial role in regulating and maintaining innate and adaptive immunity in vitiligo. Further studies on tissue/cell type-specific UPR are warranted to provide more comprehensive evidence-based support for charting the panorama of vitiligo pathogenesis.

## Abnormalities of glucose metabolism implicated in vitiligo

Various glucose pathways defects have been implicated in vitiligo. Clinical investigations can also reveal a significantly increased risk of diabetes in patients with vitiligo. In this regard, Studies by Mubki, Gopal et al. described a higher prevalence of elevated fasting glucose levels in patients with vitiligo. They concluded that the prevalence of diabetes in patients with vitiligo was statistically significant (77, 78). Several studies have also found an increased prevalence of vitiligo in patients with type II diabetes (79, 80).

It is known that pro-inflammatory cytokines play a significant role in the development of various autoimmune diseases. Pro-inflammatory cytokines such as tumor necrosis factor alpha (TNF-α), interleukin-6 (IL-6), interleukin-8 (IL-8), interleukin-1β (IL-1β), IFN-γ, and some anti-inflammatory cytokines such as interleukin-5 (IL-5) and IL-10 are increased in patients with active vitiligo (81). Inflammatory cytokines are involved in inhibiting the insulin signaling pathway by phosphorylating serine residues of the insulin receptor substrate-1, leading to insulin resistance development in vitiligo (82). Pietrzak et al. showed that impaired secretion of TNF-α, IL-6, and monocyte chemokines during glucose metabolism contributes to the induction of insulin resistance and other metabolic complications (83). We have known that CD4^+^ and CD8^+^ T cells are present at the epidermal and dermal junctions near vitiligo lesions, and CD8^+^ T cells have been proven to induce melanocytes loss, underscoring the role of T cell-mediated immunity in the pathogenesis of vitiligo (84). Recognizing imbalanced and dysfunctional effector T cell subsets and dysregulated cytokine production may have significant potential for targeted therapy. However, much remains to be explored about the mechanisms underlying dysregulated cytokine production in the pathogenesis of vitiligo.

*In vitro* studies have shown that epidermal vitiligo melanocytes have lower adenosine Triphopsphate (ATP), increased proton leakage, and altered expression of several glycolytic enzymes (hexokinase II, pyruvate dehydrogenase kinase 1, and pyruvate kinase M2) compared to healthy cells. Moreover, the defective ATP production is easily compensated by the increased activity of enzymes in glucose utilization, providing the cell with alternative substrates but not increasing energy levels but rescuing the affected activities and pathways by stabilizing the mitochondrial membrane lipid components (54). Furthermore, melanocytes in vitiligo patients are more sensitive to G6PD inhibition than normal melanocytes. A study in the Gujarat population showed a genetic and biochemical association of the G6PD 3’UTR rs1050757 polymorphism with vitiligo (85). In addition to melanocytes, keratinocytes play an essential role in oxidative stress and glycolysis processes. In a study of metabolic processes in vitiligo lesioned skin, researchers quantitatively assessed enriched metabolic pathways using gene scoring analysis. They found that Oxidative phosphorylation (OXPHOS) and glycolysis showed the most significant differences between stressed keratinocytes and the other, respectively (86). Therefore, abnormal glucose metabolism in vitiligo patients may result from an imbalance in the secretion of pro-inflammatory factors, low oxidative stress, or impaired G6PD levels.

## Abnormal lipid metabolism with oxidative stress, ferroptosis and T cell dysfunction

Previous studies on a small number of patients have reported conflicting results regarding the association between vitiligo and dyslipidemia. For example, recent findings suggest that patients with vitiligo have significant metabolic disturbances in elevated cholesterol (Chol), level of triglyceride (TG), low-density lipoprotein (LDL), and leptin levels. High-density lipoprotein (HDL) levels were lower compared to healthy controls (1). However, another study of children with vitiligo found that girls with vitiligo had lower LDL and HDL levels than controls (87). Evidence of the relationship between vitiligo and abnormal lipid metabolism is rapidly growing in the literature (2, 3, 82).

Lipids are the main components of cellular membranes, which have a highly relevant role in oxidative stress. In addition, dyslipidemia and other traditional risk factors for atherosclerosis, such as diabetes, hypertension, and smoking, activate the NADPH oxidase system, leading to excess superoxide anion production and promoting oxidative stress (88). Pietrzak et al. firstly hypothesized a role for lipid peroxidation in the pathogenesis of vitiligo (89). A study by Karadag et al. showed that hyperhomocysteinemia, except a known cardiovascular risk factor, may also contribute to the development of vitiligo by inhibiting tyrosinase (90). Reported significantly higher homocysteine levels in patients with active vitiligo compared with patients with stable vitiligo, which may induce oxidative stress, ER stress, and proinflammatory cytokine expression (91). Notably, N-homocysteinylated proteins formed by N-linkage of the carboxyl group of homocysteine with the E-amino group of lysine residues of proteins may be considered neo-antigen formation, thereby inducing the destruction of melanocytes by CD8^+^ T cell-mediated autoimmune reactions (92).

Ferroptosis, characterized by an iron-dependent increase in oxidative stress and lipid peroxidation, has been extensively studied in various diseases (93–95). However, whether ferroptosis plays a role in melanocyte loss of vitiligo remains to be elucidated. Lipid peroxide accumulation may be fatal to the cells as an integral event in ferroptosis (96). In the analysis of epidermal tissues, it was shown that ferroptosis markers were aberrantly expressed in vitiligo patients, with glutathione peroxidase 4 (GPX4) and ferritin being downregulated at both protein and mRNA levels. In contrast, transferrin receptor protein 1 (TfR) was observed to be overexpressed in both lesional and non-lesional areas of vitiligo. In addition, the same study also showed that the treatment of erastin (an inducer of iron toxicity) decreased GPX4 and ferritin levels in human epidermal melanocytes (HEMs) while increasing TfR and Acyl-CoA synthetase long-chain family member 4 (ACSL4) expression, indicating that HEMs are sensitive to ferroptosis (97). Together, ferroptosis and melanocyte loss are characterized by mitochondrial malformations, glutathione peroxidase inactivation, p53 elevation, ROS accumulation, and lipid peroxidation (98, 99). Furthermore, ferroptosis is involved in IFN-γ- associated melanocyte destruction, whereas the role of IFN-γ in vitiligo pathogenesis has been demonstrated. However, whether ferroptosis is involved in melanocyte destruction is unknown (100, 101). As a critical cell death process, Ferroptosis may provide novel therapeutic targets and serve as a potential biomarker for vitiligo.

Abnormal lipid metabolism may also contribute to T cell dysfunction in vitiligo. Compared to effector T cells and other memory T cells, tissue-resident memory T cells (T_RM_) exhibit a unique metabolic panorama. It has been reported to play an essential role in the pathogenesis of host antimicrobial infections, cancer immunotherapy, and several autoimmune diseases (102–106). Currently, the focus on T_RM_ cells may help explain the recurrence of vitiligo lesions at the same positions, as T_RM_ cells provide rapid local defense against recurrent pathogens.

Studies in vitiligo have shown that the skin surrounding vitiligo lesions is enriched with CD8^+^ T_RM_ populations expressing CD69 and CD103 during the stable and active phase (101, 107). T_RM_ cells produce perforin, Granzyme B, and IFN-γ upon interleukin-15 (IL-15) stimulation and maintain disease in cooperation with recirculating memory T (TRCM) cells. In addition, T_RM_ cells also secrete CXCL9 and CXCL10, which bind to CXCR3 on TRCM cells to recruit them to the skin. In addition, using a well-established model of CD8^+^ T_RM_ production in the skin following cowpox virus skin immunization, researchers found that cutaneous CD8^+^ T_RM_ utilized exogenous free fatty acid (FFA) internalized from the surrounding microenvironment to ensure their survival (108). This phenomenon suggested that T_RM_ cells may establish a link between lipid metabolism and adaptive memory immunity. The above studies suggest that there may be an essential target for controlling vitiligo recurrence in modulating lipid metabolism to eliminate T_RM_ in peripheral tissues.

## New therapeutic strategies in vitiligo immunometabolism

**Figure 2:**
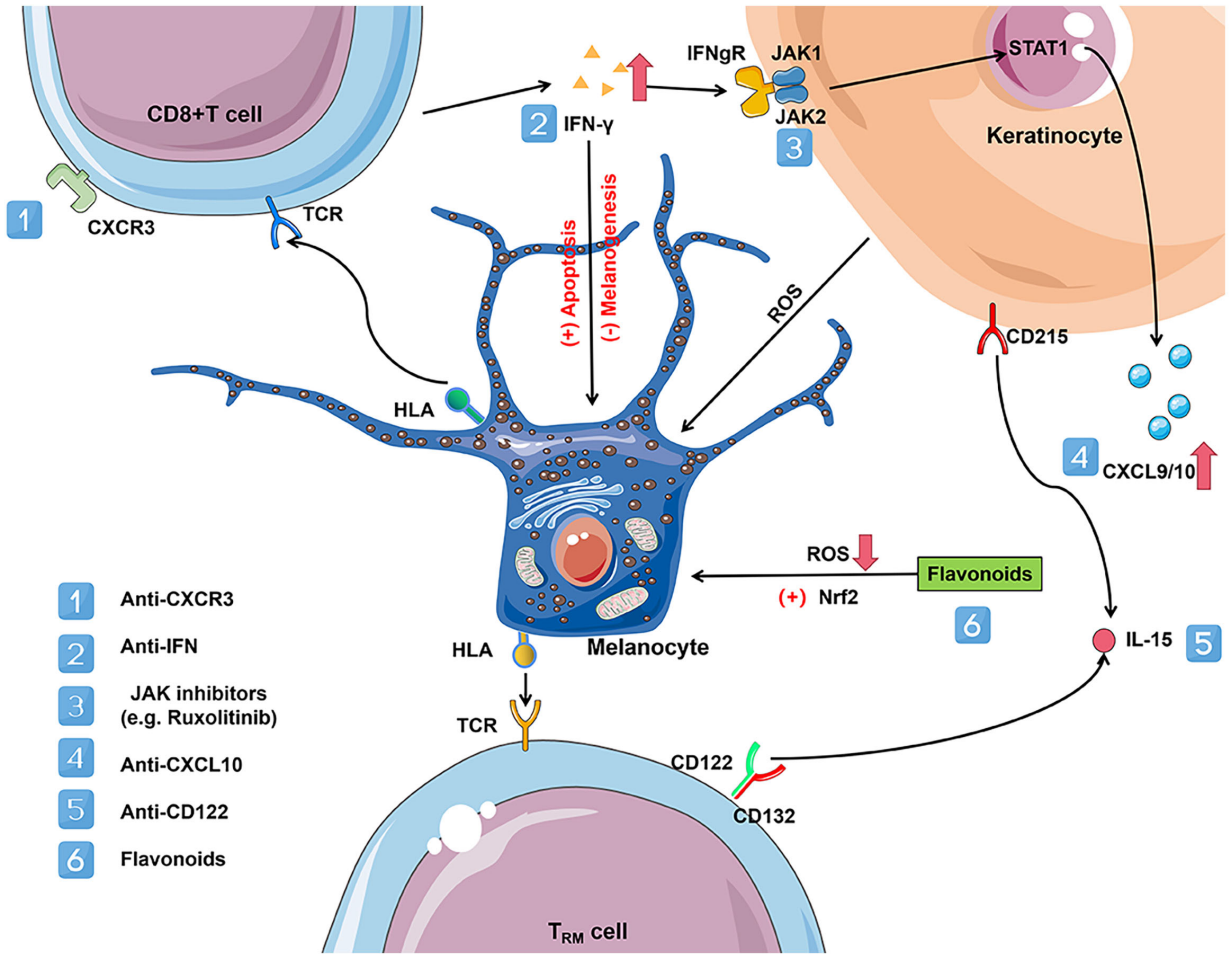
Vitiligo immunometabolism promising targets. CD8^+^ T cells in vitiligo lesions can produce a variety of cytokines, including IFN-γ. Elevated levels of IFN-γ disrupt glucose metabolism and promote melanocyte apoptosis. IFN-γ binds with IFNgR, activates the JAK-STAT pathway, and leads to the secretion of CXCL9 and CXCL10 in the skin. JAK-STAT signaling pathway activation plays a vital role in various metabolic disorders and can be treated with JAK inhibitors (e.g. ruxolitinib) acting on JAK1 and JAK2. CXCL9 promotes massive recruitment of melanocyte-specific CD8^+^ T cells to the skin *via* the cognate receptor CXCR3, while CXCL10 promotes their localization within the epidermis and their effector functions, which increases inflammation through a positive feedback loop. Activation of CXCR3B by CXCL10 induces apoptosis of melanocytes. However, depleting antibodies acting on CXCR3 could reduce the number of C8+ T cells, thereby reversing the disease. In addition, flavonoids act by activating the Nrf2/ARE signaling pathway and inhibiting NFκB activation by reducing the extent of oxidative stress in melanocytes. Established vitiligo lesions are maintained by melanocyte-reactive T_RM_ cells that maintain longevity in the skin *via* IL-15-dependent survival signals. T_RM_ is linked to lipid metabolism due to their reliance on exogenous free fatty acid (FFA) uptake to maintain their residence in the skin. The use of antibodies against CD122, the β subunit of the IL-15 receptor, in a mouse model of vitiligo effectively inhibits T_RM_ production. IL-15 and its receptor may be a target for vitiligo treatment. CXCR3, C-X-C motif chemokine receptor 3; CXCL9/10, C-X-C motif chemokine ligand 9/10; IFN-γ, inteferon-γ; IL-15, interleukin-15; IFNgR, inteferon-gamma receptor; T_RM_, resident memory T cells; TCR, T cell receptor; HLA, human leukocyte antigen; STAT1, signal transducer and activator of transcription protein 1; JAK, janus kinases.

Uncovering the biological mediators and the molecular mechanisms of metabolic defects in melanocyte degeneration and autoimmunity is essential for novel therapeutic targets and drugs intercepting the process of vitiligo. The experience with systemic biological therapies for psoriasis suggests that a similar approach might be successfully used in vitiligo. Promising treatments targeting the IFN-γ chemokine axis, JAK-STAT pathway, and Nrf2-ARE pathway, have recently emerged (**Figure 2**).

### The IFN-γ chemokine axis

The reduction in the level of IFN-γ, a key cytokine of the immune system in glucose metabolism, can improve glucose metabolism, as demonstrated in a mouse model with low IFN-γ levels (109). Furthermore, according to functional studies in a mouse model, IFN-γ, inteferon-gamma receptor (IFNgR), signal transducer and activator of transcription protein 1 (STAT1), C-X-C motif chemokine ligand 10 (CXCL10), and C-X-C motif chemokine receptor 3 (CXCR3) are also critical for developing hypopigmentation in vitiligo (46, 107, 110). Targeting CXCR3 with depleting antibodies in a mouse model reduced the number of self-reactive T cells and reversed vitiligo manifestations (111). Functional studies using conditional STAT 1 knockout mice have shown that keratinocyte-derived chemokines and IFN-γ signaling drive vitiligo and proper homing of auto-reactive T cells to the epidermis. In contrast, epidermal immune cells, such as endogenous T cells, Langerhans cells, and γδ T cells, are not required (112). IFN-γ inhibits melanogenesis and directly induces melanocyte apoptosis (113). A recent single-cell sequencing study of vitiligo showed that fibroblast pairs from different sites IFN-γ responsiveness determine its ability to recruit CD8^+^ T cells. The study also demonstrates that the degree of upregulation of CXCL9 and CXCL10 was higher in the high-incidence area. By the Cre-loxP system, they constructed an IFN-γ receptor knockout mice which no longer have vitiligo (114). Thus, the IFN- γ axis or potential targets for treating vitiligo.

### JAK-STAT pathway

In recent years, evidence has been increasing that abnormalities in the JAK-STAT pathway led to metabolic disorders. Janus kinase 2 (JAK2) is associated with central obesity and increased waist circumference (115). Mice lacking janus kinase 3 (JAK3) exhibit metabolic disorders such as insulin resistance, weight gain, increased fasting insulin and glucose levels, decreased glucose tolerance, and hepatic steatosis (116). Recent reports suggest that JAK inhibitors have good therapeutic promise in vitiligo. A recent case report found that a JAK inhibitor successfully improved glucose levels in a 19-year-old patient with type I diabetes (117). Clinical Trials of topical JAK inhibitor Ruxolitinib cream met the primary endpoint in both pivotal phases 3 clinical trials with critical secondary endpoints, with a significantly higher proportion of the trial group achieving at least 75% improvement in the F-VASI score (a quantitative measure to assess skin symptoms in patients with vitiligo) relative to baseline than the placebo group at week 24 of treatment (118). Ruxolitinib cream is currently the first Food and Drug Administration (FDA) approved vitiligo therapy for the topical treatment of non-segmental vitiligo in adults and pediatric patients over 12 years of age (119). In addition, Shiu et al. implies that combining therapies that reverse defects in keratinocytes’ metabolism with JAK inhibitors could be a novel approach to treating vitiligo (86).

### Nrf2-ARE pathway

In vitiligo, inactive Nrf2 signaling is ineffective in protecting HEM from H_2_O_2_-induced OS damage and, therefore, rarely prevents melanocyte apoptosis by mediating its downstream antioxidant gene heme oxygenase-1 (HO-1) (120). Nuclear factor (erythroid-derived 2)-like 2 (Nrf2) activation has emerged as an important target for protection against OS-related skin diseases such as skin photodamage, and vitiligo. Nuclear factor kappa-light-chain-enhancer of activated B cells (NFkB) is a significant regulator of pro-inflammatory responses and is considered a redox-regulated transcription factor that can be activated by H_2_O_2_ (121). By catalyzing the phosphorylation and degradation of IkB at specific amino acid residues, NFkB can translocate from the cytoplasm to the nucleus and further activate immune mediators such as IL-6 and IL-8 (122). These NFκB-regulated inflammatory factors accelerate islet β-cell apoptosis, cause insulin resistance in peripheral cells, and play an essential role in the pathogenesis of diabetes (123). However, the initiation of the Nrf2/ARE pathway can effectively inhibit NFκB activation. In addition, Nrf2 can inhibit the expression of TNFα, inducible nitric oxidesynthase (iNOS), and cyclooxygenase-2 (COX2) by suppressing pro-inflammatory genes. Among them, HO-1 can significantly inhibit TNFα activity and suppress the phosphorylation of the NFκB pathway promoter nuclear factor kappa-B inhibitory protein kinase (IKK)β, thereby suppressing inflammation (21). NFκB can also counteract the transcriptional activity of Nrf2 and suppress the expression of HO-1 (124). Flavonoids such as Ginkgo biloba extract EGb761 and afzelin can protect melanocytes from OS-induced apoptosis by enhancing the Nrf2 signaling pathway and its downstream antioxidant (125, 126).

### Tissue-resident memory cell target

T_RM_ is inextricably linked to lipid metabolism due to its dependence on exogenous FFA uptake to maintain its residency in the skin. Functional CD8^+^ T_RM_ has been found in both stable and active vitiligo, suggesting that those cells maintained in stable disease could explain disease reactivation (127). Thus, intercepting critical metabolic pathways that deplete T_RM_ may be promising, such as trimetazidine, which blocks mitochondrial β-oxidation of FFA and reduces T_RM_ lifespan in the skin (108, 128). In preclinical mice models of vitiligo, the use of antibodies against CD122 (the β-subunit of the IL-15 receptor, expressed on T_RM_ cells) effectively inhibits T_RM_ production. IL-15 and its receptor might be a promising target for vitiligo treatment (129). However, the precise molecular mechanisms involved in this phenomenon and the putative clinical implications of this T-cell regulation remain to be determined, as some discrepancies exist between *in vivo* and *in vitro* findings.

In addition to their beneficial effects on the primary and secondary prevention of cardiovascular events, lipid-lowering agents statins may also attenuate vitiligo disease activity. As an inhibitor of the pro-inflammatory cytokine, they protect against H_2_O_2_-induced apoptosis and ROS accumulation. In addition, they inhibit CD8^+^ T cell action in melanocytes through IFN-γ and even block the T cells involved in the T generation of the PI3K/AKT signaling pathway (130–132).

Due to significant metabolic alterations in tumor cells, oncologists have long expected to treat tumors by inhibiting upregulated metabolic pathways, but the actual effects have been suboptimal. Compared to the tumor cell that needs to be cleared entirely, the normal number and proportion of immune cells still need to be maintained to keep immune homeostasis in autoimmune diseases. However, multiple energy metabolism is altered in autoimmune disease pathogenesis, increasing treatment difficulty. Since immunometabolism research in vitiligo is still unclear, there is still much to be discovered about related treatments, and their therapeutic effects need to be confirmed by more studies.

## Conclusion and future perspective

Immunometabolism has emerged as a major and popular area of research in immunological disease, focusing on cellular metabolism and metabolic programs of specific cells, have advanced our understanding of pathogenesis and novel therapeutic targets. Although substantial progress has been made in the pathogenesis of vitiligo, there are still many questions remain to be answered. Immunometabolism in the pathogenesis of vitiligo is one of the most attractive research areas, which raised by the melanocytes from vitiligo patients are more susceptible to oxidative stress and finally lead to activation of the innate immune response and subsequently to adaptive immune response through activation of autoreactive cytotoxic CD8^+^ T cells. However, the mechanisms of how immunometabolism change skin melanocyte and immune cell infiltrations and function are not sufficiently explored and reviewed.

Here in this study, we reviewed the oxidative stress related physiological progress and specific defects in the metabolic pathways, which promote dysregulation of innate and adaptive immune responses in vitiligo. These abnormalities appear to be driven by genetic and epigenetic factors modulated by stochastic events. In addition to extensive descriptions of abnormalities in immunometabolism of vitiligo, recent studies support the critical role of dysregulation of metabolic pathways in various skin immune cells, including keratinocytes, tissue-resident memory T cells, and innate lymphoid cells of vitiligo pathogenesis.

Identifying therapeutic targets is a key goal for the research field. Currently, treatments targeting the IFN-γ chemokine axis, JAK-STAT pathway, and Nrf2-ARE pathway, have been recently emerged and reviewed. This review summarizes the current knowledge on immunometabolism reprogramming in the pathogenesis of vitiligo and gives an overview of future vitiligo treatment. Future studies may incorporate the effect of the microenvironment on immune cell metabolism and finding new approaches to understand the diversity of metabolic programs in specific cell population and ultimately for discovery of immune modulation and novel therapeutic targets.

## Author contributions

CL drafted and edited the manuscript; YS edited and approved the final version of the manuscript. All authors contributed to the article and approved the submitted version.

## Funding

This work was supported by the Taishan Scholars Program of Shandong Province (tsqn201909141), Shandong Provincial Youth Science and Technology Talents Support Plan (ZR2020YQ56), the National Natural Science Foundation of China (81502736,82073441, 81874244).

## Conflict of interest

The authors declare that the research was conducted in the absence of any commercial or financial relationships that could be construed as a potential conflict of interest.

## Publisher’s note

All claims expressed in this article are solely those of the authors and do not necessarily represent those of their affiliated organizations, or those of the publisher, the editors and the reviewers. Any product that may be evaluated in this article, or claim that may be made by its manufacturer, is not guaranteed or endorsed by the publisher.
